# The effect of eight weeks resistance and aerobic training on myostatin and follistatin expression in cardiac muscle of rats

**DOI:** 10.15171/jcvtr.2016.33

**Published:** 2016-12-30

**Authors:** Amir Rashidlamir, Seyyed Reza Attarzadeh Hosseini, Keyvan Hejazi, Seyyed Mohamad Motevalli Anberani

**Affiliations:** ^1^Faculty of Physical Education and Sport Sciences, Ferdowsi University of Mashhad, Mashhad, Iran; ^2^Professor in Sport Physiology, Faculty of Physical Education and Sports Sciences, Ferdowsi University of Mashhad, Mashhad, Iran; ^3^PhD Student of Physical Education and Sport Sciences, Ferdowsi University of Mashhad, Mashhad, Iran

**Keywords:** Myostatin Gene Expression, Follistatin, Resistance Exercise, Aerobic Exercise, Myocardium Muscle

## Abstract

***Introduction:*** The clinical studies have shown that the myostatin gene expression and its serum density occur more frequently in heart patients than in healthy individuals. The purpose of this study is to investigate the influence of 8-week resistance and aerobic exercise on the myostatin and follistatin gene expression of myocardium muscle of healthy male Wistar rats.

***Methods:*** In this experimental study, 20 five-week-old adult Wistar rats (250 ± 26.5 g) were divided into three groups: healthy control group (n = 6), resistance exercise group (n = 7), and aerobic exercise group (n = 7). The resistance and aerobic exercise plan consisted of 8 weeks and 3 sessions per week. The resistance exercise group performed climbing a one-meter 26-stair ladder with a slope of 85 degrees for 3 sets of 5 repetitions per session. The aerobic exercise group performed running at a speed of 12 meters per minute for 30 minutes during the first sessions gradually increasing up to a speed of 30 meters per minute for 60 minutes during the final sessions (equivalent to 70% to 80% of maximum oxygen consumption). The differences between the groups were evaluated using a one-way analysis of variance (ANOVA) test. When appropriate, LSD post-hoc test was used. The significance level for the study was less than 0.05.

***Results:*** The results of this study shows that after 8 weeks of exercise, there is no significant difference between myostatin mRNA gene expression levels of the heart muscle among the three groups of control, resistance exercise, and aerobic exercise (*P* = 0.172, F = 1.953). However, the mean differences between follistatin mRNA levels of the heart muscle among the three groups of control, resistance exercise, and aerobic exercise are statistically significant (F = 38.022, *P* = 0.001). Furthermore, the ratio of follistatin to myostatin mRNA gene expression of the heart muscle (*P* = 0.001, F = 10.288) shows significant difference among the three groups.

***Conclusion:*** Our results indicate that the resistance and aerobic exercise could cause a decrease in myostatin and an increase in follistatin levels, thus preventing many muscular physiological disorders such as arthritis and muscle weakness.

## Introduction

The clinical studies have shown that the myostatin gene expression and its serum density occur more frequently in heart patients as compared with healthy individuals.^1^ Myostatin gene expression increases within the periods of skeletal muscle inactivity and/or the prevention of serum myostatin leads to the building of strength and muscle mass.^2,3^ Myostatin is a protein produced by skeletal muscle cells which penetrates into the blood of living cells and inhibits the muscle growth.^4^

The growth and differentiation myostatin/factor 8 has been introduced as a factor causing muscle weakness.^5^ The myostatin gene is located in the centromeric region of chromosome 2 and contains three exons and two interferons in the lower area of the gene.‏ The myostatin gene acts as mediator of the gene expression in relation to the control of the fiber muscle formation, and basically inhibits the muscle growth through the prevention of myoblast proliferation.^6^ This activity of myoblast is mainly related to the growth of prenatal muscles during the myoblast proliferation and differentiation period.^7^ In this sense, the activity of myostatin can be influenced by other interactive factors such as follistatin, the pseudo gene of follistatin, the serum protein associated with the growth and differentiation factor, and the myostatin receptor (activin IIb).^8^ The most significant role of follistatin, a glycoprotein nearly expressed in all tissues of mammals, is to neutralize the activity of TGF-β family proteins such as myostatin. In the presence of follistatin, myostatin is not able to connect to its own receptor and this can prevent the muscular dystrophy caused by myostatin.^8,9^ Myostatin expressed during periods of inactivity increases skeletal muscle^10^ or the inhibition of serum myostatin increases strength and muscle mass.^11^ Therefore, it seems that the resistance training leads to decreased expression of myostatin.^12^ According to the findings, the myostatin gene expression in the heart muscle can change following the physical activity or the myocardial infarction. These changes are so important and influential that can also affect the skeletal muscles, causing them to atrophy.^13^

However, considering the importance of physical activity in the prevention and treatment of many diseases, specialists suggest the exercise and nutritional counseling to treat cardiovascular diseases prior to drug therapy. In addition, exercise performance causes more satisfaction and pleasure as compared with therapeutic and drug regimens. In this sense, on one hand, the study seems to be important because the results of this study maybe used to help in the treatment and non-drug rehabilitation of some diseases such as heart failure and abnormal thickening of the heart muscle. On the other hand, determining the role of myostatin in the mechanism of cardiac adaptations following the resistance and aerobic exercises can offer a new position for myostatin in exercise sciences. Now, given the fact that the simultaneous effect of resistance and aerobic exercises is not emphasized as just aerobic exercises on the myostatin and follistatin gene expression levels, which are considered as one of cardiovascular risk factors, and that there is still some uncertainty in the limited studies carried out regarding the intervention of resistance and aerobic exercises in reducing the expression of this gene. The present study thus aimed at exploring the influence of 8-week resistance and aerobic exercise program on the myostatin and follistatin gene expression in the heart muscles of male Wistar rats.

## Materials and Methods

### Subjects

In this experimental study, 20 five-week-old adult Wistar rats (250 ± 26.5 g) were divided into three groups: healthy control group (n = 6), resistance exercise group (n = 7), and aerobic exercise group (n = 7). The rats were placed in an animal house under the laboratory conditions for 2 weeks (temperature between 20 and 22°C with the 12 hour light/dark cycles). The rats stayed and were kept in Plexiglas cages with perforated doors and fed on special food for rodents. Likewise, the water was provided by a special glass bottle and their cages were disinfected with 70% alcohol 3 times a week.

### The training program

#### The familiarization phase and resistance exercise

The resistance and aerobic exercise plan consisted of 8 weeks and 3 sessions per week. After a week of familiarization with the laboratory environment, the rats were familiarized with the way of climbing a ladder with a weight equivalent to the 30% of body weight of the animal for 10 to 15 minutes through a cylinder which was attached to its tail. The resistance exercise group performed climbing a one-meter 26-stair ladder with a slope of 85 degrees for 3 sets of 5 repetitions per session. The rest interval between the sets was two minutes while it was 1 minute between the repetitions. The way of adding weight was that the amount of weight strapped to the rats in the first week equaled the 30% of their body weight which gradually increased to almost 200% of their body weight in the last 2 weeks.

#### Aerobic exercises

In this study, the aerobic exercise group performed running at a speed of 12 m/min for 30 minutes during the first sessions gradually increasing up to a speed of 30 m/min for 60 minutes during the final sessions (equivalent to 70% to 80% of maximum oxygen consumption).^14^

### Biopsy and variable measurement

Twenty-four hours after the last training session and 12 hours after fasting, the rats in all groups were sacrificed after transferring to the genetic laboratory and their muscle tissues were used as samples to estimate the levels of myostatin mRNA and follistatin.^15^ After anesthetizing and fixing the animals on the board of rodent surgery, the autopsy was performed. The muscle tissue samples were taken immediately after the autopsy, the samples were then taken from the left ventricle of the rats. And the 10% formalin was placed in fixative and was kept in the solution for 48 hours. After the first 24 hours, the new formalin was replaced with the previous formalin. After fixation with dewatered alcohol, it was molded with paraffin. After this process, the microtome sections with 5 micron thickness were taken through random sampling at regular intervals and then were examined. To investigate the expression of myostatin mRNA and follistatin in the heart muscle, the RT-PCR method with primer sequences was used. Myostatin primer included: Forward primer: 5’-TAA CCT TCC CAG GAC CAG GA-3’ and Reverse primer: 5’-CAC TCT CCA GAG CAG TAA TT-3’ and follistatin primer included forward primer: 5’-CAG TGC AGC GCT GGA AAG AAA T-3’ and reverse primer: 5’-TGC GTT GCG GTA ATT CAC TTA C-3’.

### RT-PCR method

For RT-PCR reaction, the Chromo device along with the diagnostic conjugate of SYBER-Green product, a commercial product of TAKARA, was used in this study. In this sense, the necessary ingredients were added to the special tubes to make the reaction occur on the genes in question at different time scales and also in upper and lower parts of the incision. To reduce the possibility of error in pouring the materials, first a Master Mix was prepared for each gene, which contained all the above ingredients except the cDNA. After the complete dissolving of the μI18 materials of Master Mix in each special real-time tube was poured and finally two micro-liters of cDNA related to each tube was individually poured.

### Statistical analysis

All values are presented as mean ± standard deviation (SD). The data collected were analyzed using SPSS 16.0 (SPSS Inc., Chicago, IL, USA). Data distribution normality and homogeneity of variance were examined with Shapiro-Wilk and Levene’s test respectively. The differences between the groups were evaluated using a one-way analysis of variance (ANOVA) test. When appropriate, LSD post-hoc test was used. The significance level for the study was less than 0.05.

## Results

### Heart muscle myostatin mRNA expression

**Figure 1:**
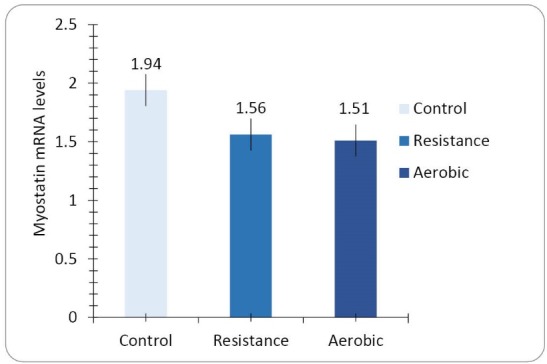
Changes in mRNA expression of myostatin levels in myocardium muscle in different groups.

The results of this study shows that after 8 weeks of exercise, there is no significant difference between myostatin mRNA gene expression levels of the heart muscle among the three groups of control, resistance exercise, and aerobic exercise (*P* = 0.172, F = 1.953, see Figure 1). LSD test results showed that there is no significant difference in heart muscle myostatin mRNA expression among the control group with resistance group (*P* = 0.127) and aerobic group (*P* = 0.084), and also between the resistance exercise and aerobic exercise groups (*P* = 0.81).

### Heart muscle follistatin mRNA expression

**Figure 2:**
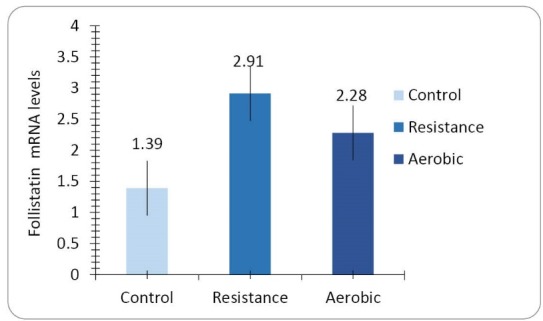
Changes in mRNA expression of follistatin levels in myocardium muscle in different groups.

**Figure 3:**
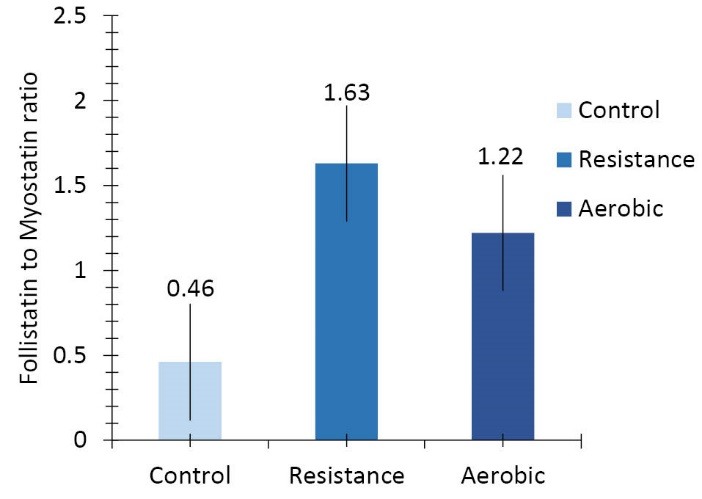
Changes in mRNA expression of follistatin to myostatin ratio levels in myocardium muscle in different groups.

The mean differences between follistatin mRNA levels of the heart muscle among the three groups of control, resistance exercise, and aerobic exercise are statistically significant (F = 38.022, *P* = 0.001, Figure 2). LSD test results showed that there is significant difference in heart muscle myostatin mRNA expression‏ between‏ the control group and resistance exercise group (*P* = 0.001) and aerobic group (*P* = 0.001), and also between the resistance exercise group and the aerobic exercise group (*P* = 0.001). The ratio of follistatin to myostatin mRNA gene expression of the heart muscle (*P* = 0.001, F = 10.288, Figure 3) shows significant difference among the three groups.

## Discussion

The aim of this study was to investigate the effect of 8 weeks of resistance and aerobic exercises on the expression of myostatin and follistatin genes of healthy male Wistar rats’ heart muscles. According to the results of the study, the resistance and aerobic exercises led to a decrease in myostatin mRNA levels in the heart muscle male Wistar rats as compared to the control group, whereas these changes were not statistically significant. However, the relationship between the inhibition of myostatin and the exercise adaptations to the size and performance of the heart muscle has not been determined. In particular, whether the myostatin inhibition affects the physiological hypertrophy of the heart muscle which is caused by the adaptation to resistance exercise?^16^ Myostatin oblast is a negative regulator of muscle growth in which if a mutation occurs in the coding area of this gene, it changes the role of its regulators and causes the muscle strength through the increase of protein synthesis.^17^ The presence of this protein influences the hormone effective in the resistance of tendons and their flexibility, and it then leads to the weakness and decrease in the flexibility quality of tendons. The transforming growth factor-beta (TGF-β) is the most important cytokine of regulating skeletal muscle growth. As a member of this group, myostatin‏ plays a crucial role in the control of muscle mass; in fact, the human-animal studies indicate the negative regulating role of myostatin in skeletal muscle growth.^18^ Given the studies on the effects of physical exercise on the rats lacking myostatin, the researchers have all argued that the inactivation of myostatin limits various aerobic functions in the rats.^19-21^

This is probably due to the high proportion of glycolytic fibers and the large fiber size in the rats lacking myostatin.^22^ However, in the older rats, the inactivation of myostatin in combination with physical exercise can improve endurance performance.^23^ In this sense, Artaza et al^24^ and Cohn et al^25^ have attempted to explore the relationship between myostatin, the heart size, and also the cardiac physiological parameters and have offered conflicting findings in terms of heart size; however, both these studies indicate that the minimum basic parameters and cardiac output (stroke volume) are normal in the hearts lacking myostatin. Having a review of research literature on this issue, we realized that other factors such as follistatin, FLRG, and GASP-1, and the myostatin receptors (activin IIb) affect the function of myostatin without influencing the process of myostatin gene expression. In this context, myostatin acts in different ways such as inhibiting the activity of satellite cells,^26^ proliferation my oblasts,^27^ and myogenic differentiation.^28^

In addition, one of the mechanisms through which myostatin suppresses muscle growth like most family members of TGF-β includes a mechanism in which myostatin (through breaking down proteins) becomes an end of amine (inactive area) and an end of carboxyl (active area) in the endoplasmic reticulum. The secreted myostatin then flows in the blood in the form of a multi-protein complex which includes a dual connection, which is not covalent, to the inactive area or other inhibitory proteins such as follistatin. The active dual myostatin of a disable protein complex is probably released through the degrading enzymes present in the extracellular matrix of muscle and other tissues.^29^

After the myostatin is released, the activin type II or IIb is then joined and leads to phosphorylation of the protein family.^30^ However, there is also evidence suggesting that myostatin can make muscle mass adjust without affecting SMAD. Regardless of this course, myostatin can prevent the proliferation^31^ and differentiation^32^ of muscle stem cells, or can weaken the growth of mature muscle fibers, which consequently causes the reduction of skeletal muscle mass to emerge.^33^

Another reason for the myostatin reduction after exercise can be related to the muscle imbalance of growth regulators toward the positive regulators. In a normal situation, there is a hemostatic balance between the important positive regulators (such as IGF-1) and the negative regulators (such as myostatin) of muscle growth in order to keep the size of muscle fibers; however, this balance leads to the negative regulators if the muscle is atrophied; and if the load is applied on the muscle, it will lead to the positive regulators. Although the mechanism of the regulators is not entirely clear, it seems that this relationship is established through a negative complex feedback loop.^34^

Myostatin in muscle cells has a dual function. On the one hand, the increase in FOX1 as one of the important cellular pathways is responsible for the increased protein degradation and ultimately apoptosis, but, on the other hand, this reduces the amount of mTOR as the most important regulator of protein synthesis within the cell. The increase of any of the positive or negative factors in the feedback loop through some factors such as PI3K, GSK3β and MuRF-1affectthe expression and secretion of myostatin in muscle cells.^35^ In this sense, one of the possible causes of reducing the amount of myostatin immediately after exercise can be related to the imbalance of the muscle growth regulators toward the positive regulators. According to the results of this study, regular aerobic and resistance exercise resulted in a significant increase in mRNA follistatin levels of Wistar rats’ heart muscle. The follistatin adjustment during sports activities is unclear, but it is important because physical activity is an important intervention for the prevention of muscular atrophy.^36^ Follistatin as an important inhibitor of myostatin expression, influenced by a very complex molecular and cellular mechanism, prevents myostatin expression. Moreover, it has been shown that follistatin can be considered as a competitive inhibitor for myostatin. In this sense, follistatin prevents myostatin from connecting to its receptor by connecting to the point of activin type IIb receptor.^37,38^

Therefore, a significant increase in follistatin through the aerobic and resistance exercises in particular, follistatin connects to myostatin receivers (activin type IIb), myostatin less than before can be connected to its receptors and their catabolic effects are less likely to leave. These anabolic effects can lead to an increase in fat-free mass in Wistar rats which then results in an increased in their performance.^36^ Follistatin probably plays a key role in reducing the myostatin signals. Thus, the observed increase in follistatin was accompanied by a decrease in myostatin in aerobic and resistance exercise groups which prevents myostatin signaling and muscle catabolism as a result. According to the previous studies, however, follistatin connects to myostatin, nullifies it, and then increases the hypertrophy and hyperplasia of the skeletal muscles.^37,39^

According to the results of this study, the regular aerobic and resistance exercise resulted in a significant increase in mRNA levels of follistatin to myostatin in the Wistar rats’ heart and skeletal muscle. According to the results of this study, the amount of follistatin decreases while the amount of follistatin values ​​increases, which seems a reasonable ratio. The resistance exercises would greatly increase the anabolic state; therefore, it seems that the implementation of resistance exercises along with aerobic exercises can lead to a decrease in the metabolic state. The reasons for this phenomenon include the neuromuscular adaptation through doing the exercises. In this sense, the balance between anabolic and catabolic responses may play an important role in the interfering effects of resistance and aerobic exercises. Thus, using resistance and aerobic exercise can lead to a reduction in myostatin and an increase in follistatin and prevents the physiological disorders of muscle such as atrophy and muscle weakness. We suggest that adaptation and alteration in mRNA levels of follistatin and myostatin gene expression in the Wistar rats’ heart and skeletal muscle depend on duration and intensity of exercise. Due to the fact that the role of genetic factors, as an important factor in the development of cardiovascular disease, is still unknown in Iran. Therefore, due to limited research on the impact of physical activity, especially resistance and aerobic training on the expression of myostatin and follistatin gene and, the effect of these exercises on the heart and skeletal muscle more researches are requires. We suggest that adaptation and alteration in mRNA levels of follistatin and myostatin gene expression in the Wistar rats’ heart and skeletal muscle depend on duration and intensity of exercise. Due to the fact that the role of genetic factors, as an important factor in the development of cardiovascular disease, is still unknown in Iran. Therefore, due to limited research on the impact of physical activity, especially resistance and aerobic training on the expression of myostatin and follistatin gene and, the effect of these exercises on the heart and skeletal muscle more researches are requires.

## Conclusion

Overall, it is clear that we need further studies to prove the actions of myostatin and follistatin, especially in the exercise sciences; therefore, the studies on the effect of resistance and aerobic exercises on mRNA expression of myostatin and follistatin are limited. Also, understanding the specificity of compatibility exercises may provide therapeutic goals in order to treat the cardiovascular diseases of heart muscles and show the most effective way to prevent or ameliorate these diseases.

## Ethical approval

This study was approved by medical ethics committee of Ferdowsi University of Mashhad.

## Competing interests

The authors declare no conflicts of interest.

## Acknowledgments

This project was supported by a grant from Ferdowsi university of Mashhad. This study was part of a project of the physiology research center (Grant No.25398). We would like to express our specific thanks to the deputy of research affairs of Ferdowsi university of Mashhad for their financial support.

## References

[1] Fernández-Solà J, Borrisser-Pairo F, Antunez E, Tobías E (2015). Myostatin and insulin-like growth factor-1 in hypertensive heart disease: a prospective study in human heart donors. J Hypertens.

[2] Agung P, Said S, Sudiro A (2016). Myostatin gene analysis in the first generation of the belgian blue cattle in indonesia. Journal of the Indonesian Tropical Animal Agriculture.

[3] Lee S-J, Lee Y-S, Zimmers TA (2010). Regulation of muscle mass by follistatin and activins. Mol Endocrinol.

[4] Casas E, Shackelford S, Keele J, Stone R, Kappes S, Koohmaraie M (2000). Quantitative trait loci affecting growth and carcass composition of cattle segregating alternate forms of myostatin. J Anim Sci.

[5] Elbialy ZI, El-Nahas AF, Elkatatny NA, Ammar AY (2016). quantitative expression analysis of myostatin gene in Nile Tilapia (Oreochromis niloticus) tissues in adult stage. Alexandria Journal of Veterinary Sciences.

[6] Shin S, Song Y, Ahn J, Kim E, Chen P, Yang S (2015). A novel mechanism of myostatin regulation by its alternative splicing variant during myogenesis in avian species. Am J Physiol Cell Physiol.

[7] Soufi B, Shojaeian K, Mohammad Abadi M, Baghi zadeh A, Ferasati S (2008). Myostatin gene polymorphism in Sanjabi sheep breed using molecular marker. J Anim Sci.

[8] Bilezikjian LM, Blount AL, Corrigan AZ, Leal A, Chen Y, Vale WW (2001). Actions of activins, inhibins and follistatins: implications in anterior pituitary function. Clin Exp Pharmacol Physiol.

[9] Hiroki E, Abe S, Iwanuma O, Sakiyama K, Yanagisawa N, Shiozaki K (2011). A comparative study of myostatin, follistatin and decorin expression in muscle of different origin. Anat Sci Int.

[10] Dominique JE, Gérard C (2006). Myostatin regulation of muscle development: molecular basis, natural mutations, physiopathological aspects. Exp Cell Res.

[11] Whittemore L-A, Song K, Li X (2003). Inhibition of myostatin in adult mice increases skeletal muscle mass and strength. Biochem Biophys Res Commun.

[12] Asad M, Vakili J (2013). Effect of 8 weeks resistance training on myostatin serum level in overweight nonathletic women. Int J Sport Stud.

[13] Heineke J, Auger-Messier M, Xu J, Sargent M, York A, Welle S (2010). Genetic deletion of myostatin from the heart prevents skeletal muscle atrophy in heart failure. Circulation.

[14] Lee S, Farrar RP (2003). Resistance training induces muscle-specific changes in muscle mass and function in rat. J Exercise Physiol Online.

[15] Lenk K, Schur R, Linke A, Erbs S, Matsumoto Y, Adams V (2009). Impact of exercise training on myostatin expression in the myocardium and skeletal muscle in a chronic heart failure model. Eur J Heart Fail.

[16] Allen DL, Cleary AS, Speaker KJ, Lindsay SF, Uyenishi J, Reed JM (2008). Myostatin, activin receptor IIb, and follistatin-like-3 gene expression are altered in adipose tissue and skeletal muscle of obese mice. Am J Physiol Endocrinol Metab.

[17] Elkina Y, von Haehling S, Anker SD, Springer J (2011). The role of myostatin in muscle wasting: an overview. J Cachexia Sarcopenia Muscle.

[18] Wang R, Jiao H, Zhao J, Wang X, Lin H (2016). Glucocorticoids enhance muscle proteolysis through a myostatin-dependent pathway at the early stage. PloS One.

[19] Matsakas A, Bozzo C, Cacciani N, Caliaro F, Reggiani C, Mascarello F (2006). Effect of swimming on myostatin expression in white and red gastrocnemius muscle and in cardiac muscle of rats. Exp Physiol.

[20] Personius KE, Jayaram A, Krull D, Brown R, Xu T, Han B (2010). Grip force, EDL contractile properties, and voluntary wheel running after postdevelopmental myostatin depletion in mice. J Appl Physiol (1985).

[21] Louis E, Raue U, Yang Y, Jemiolo B, Trappe S (2007). Time course of proteolytic, cytokine, and myostatin gene expression after acute exercise in human skeletal muscle. J Appl Physiol.

[22] Schiffer T, Geisler S, Sperlich B, Strüder H (2011). MSTN mRNA after varying exercise modalities in humans. Int J Sports Med.

[23] LeBrasseur NK, Schelhorn TM, Bernardo BL, Cosgrove PG, Loria PM, Brown TA (2009). Myostatin inhibition enhances the effects of exercise on performance and metabolic outcomes in aged mice. J Gerontol A Biol Sci Med Sci.

[24] Artaza JN, Reisz-Porszasz S, Dow JS, Kloner RA, Tsao J, Bhasin S (2007). Alterations in myostatin expression are associated with changes in cardiac left ventricular mass but not ejection fraction in the mouse. J Endocrinol.

[25] Cohn RD, Liang H-Y, Shetty R, Abraham T, Wagner KR (2007). Myostatin does not regulate cardiac hypertrophy or fibrosis. Neuromuscul Disord.

[26] McCroskery S, Thomas M, Maxwell L, Sharma M, Kambadur R (2003). Myostatin negatively regulates satellite cell activation and self-renewal. J Cell Biol.

[27] Thomas M, Langley B, Berry C, Sharma M, Kirk S, Bass J (2000). Myostatin, a negative regulator of muscle growth, functions by inhibiting myoblast proliferation. J Biol Chem.

[28] Ríos R, Carneiro I, Arce VM, Devesa J (2002 May). Myostatin is an inhibitor of myogenic differentiation. Am J Physiol Cell Physiol.

[29] Wolfman NM, McPherron AC, Pappano WN, Davies MV, Song K, Tomkinson KN (2003). Activation of latent myostatin by the BMP-1/tolloid family of metalloproteinases. Proc Natl Acad Sci U S A.

[30] Rebbapragada A, Benchabane H, Wrana J, Celeste A, Attisano L (2003). Myostatin signals through a transforming growth factor β-like signaling pathway to block adipogenesis. Mol Cell Biol.

[31] Taylor WE, Bhasin S, Artaza J, Byhower F, Azam M, Willard DH Jr (2001). Myostatin inhibits cell proliferation and protein synthesis in C2C12 muscle cells. Am J Physiol Endocrinol Metab.

[32] Langley B, Thomas M, Bishop A, Sharma M, Gilmour S, Kambadur R (2002). Myostatin inhibits myoblast differentiation by down-regulating MyoD expression. J Biol Chem.

[33] Hosseini SA, Rad M, Hejazi K (2016). Effects of Ramadan fasting and regular physical activity on serum myostatin and follistatin concentrations. International Journal of Applied Exercise Physiology.

[34] Lee SJ, McPherron AC (2001). Regulation of myostatin activity and muscle growth. Proceedings of the National Academy of Sciences.

[35] Favier FB, Benoit H, Freyssenet D (2008). Cellular and molecular events controlling skeletal muscle mass in response to altered use. Pflugers Arch.

[36] Hansen J, Brandt C, Nielsen AR, Hojman P, Whitham M, Febbraio MA (2011). Exercise induces a marked increase in plasma follistatin: evidence that follistatin is a contraction-induced hepatokine. Endocrinology.

[37] Dieli-Conwright CM, Spektor TM, Rice JC, Sattler FR, Schroeder ET (2009). Influence of hormone replacement therapy on eccentric exercise induced myogenic gene expression in postmenopausal women. J Appl Physiol.

[38] Rodino‐Klapac LR, Haidet AM, Kota J, Handy C, Kaspar BK, Mendell JR (2009). Inhibition of myostatin with emphasis on follistatin as a therapy for muscle disease. Muscle Nerve.

[39] Aoki MS, Soares AG, Miyabara EH, Baptista IL, Moriscot AS (2009). Expression of genes related to myostatin signaling during rat skeletal muscle longitudinal growth. Muscle Nerve.

